# Right atrial volume measured by cardiac magnetic resonance correlates with NT-ProBNP and invasive right atrial pressure in pulmonary hypertension, with and without systemic sclerosis

**DOI:** 10.1186/1532-429X-18-S1-P300

**Published:** 2016-01-27

**Authors:** Tom Gyllenhammar, Katarina Steding-Ehrenborg, Marcus Carlsson, Göran Rådegran, Roger Hesselstrand, Håkan Arheden, Ellen Ostenfeld

**Affiliations:** 1 Dept of Clinical Physiology, Cardiac MR Group, Lund, Sweden, Sweden; 2 grid.4514.40000000109302361Department of Clinical Sciences Lund, Lund University, and Lund University Hospital, Lund, Sweden; 3 grid.4514.40000000109302361Department of Health Sciences, Lund University, Physiotherapy, Lund Sweden; 4 The Section for Heart Failure and Valvular Disease, Lund, Sweden; 5 Department of Rheumatology, Lund, Sweden

## Background

Right atrial (RA) pressure (RAP) and NT-ProBNP-levels are important prognostic factors in pulmonary hypertension (PH). The aim of this study was to investigate if RA volume (RAV), emptying fraction and emptying volume measured with cardiac magnetic resonance (CMR) can be used to predict RAP, and to investigate if these measures are related to NT-ProBNP levels. Furthermore, we aimed to determine if RAV in systemic sclerosis patients with precapillary PH (PH_SSc_) differs from PH patients without systemic sclerosis (PH_nonSSc_).

## Methods

We included 27 patients with PH (54 ± 19 years, 18 women). PH was defined as mPAP ≥25 mmHg and PCWP ≤15 mmHg at normal or reduced cardiac output. 11 patients with and 16 patients without Systemic Sclerosis and 35 healthy controls (age 31 ± 9 years, 16 women) underwent cine CMR to quantify end-systolic maximum (RAV_max_) and end-diastolic minimum (RAV_min_) right atrial volume indexed to body surface area. Invasive pressures were measured with right heart catheterization and plasma NT-ProBNP level from venous blood samples.

## Results

In all PH patients (PH_SSc_ and PH_nonSSc_) mRAP was 7 ± 6 mmHg, sPAP 73 ± 23 mmHg, mPAP 46 ± 16 mmHg and PCWP 8 ± 4 mmHg. The correlation coefficient (r) between mRAP and RAV_min_ was 0.46 (p=0.015) and between mRAP and RAV_max_ 0.43 (p=0.024). Mean NT-ProBNP was 1894 ± 2381 ng/L. In the PH patients, mRAP correlated with NT-ProBNP (r = 0.5, p = 0.019). There was a strong correlation between NT-ProBNP and RAV_min_ (r = 0.7, p = 0.0003) and RAV_max_ (r = 0.67, p = 0.0006).

RAV_min_ in patients with PH_nonSSc_ (97 ± 35 ml/m^2^) was higher as compared to PH_SSc_ (54 ± 23 ml/m^2^, p < 0.05) and healthy controls (57 ± 12 ml/m^2^, p < 0.05), but did not differ between patients with PH_SSc_ and healthy controls (NS). There was also a significant difference in RAV_max_ between the patients with PH_nonSSc_ (69 ± 32 ml/m^2^) and PH_SSc_ (32 ± 19 ml/m^2^, p < 0.05) as well as healthy controls (24 ± 8 ml/m^2^, p < 0.05), yet no difference between PH_SSc_ and healthy controls (NS). RA emptying fraction differed between the PH_nonSSc_ patients and the healthy controls (31 ± 12% vs. 54 ± 15%, p < 0.05), but neither between PH_SSc_ patients (43 ± 18%) and healthy controls nor PH patients (ns). There was no significant difference in RA emptying volume between the groups PH_nonSSc_ (29 ± 13 ml/m2), PH_SSc_ (21 ± 11 ml/m^2^) and the healthy controls (31 ± 10 ml/m^2^).

## Conclusions

This study shows that non-invasive measures of right atrial volumes by cardiac magnetic resonance correlates with NT-ProBNP and invasive right atrial pressure in patients with precapillary PH. Furthermore, RAV_min_ and RAV_max_ were increased in PH_nonSSc_ compared to PH_SSc_. Future studies are needed to investigate the clinical advantages of these complementary measures in the diagnostics of pulmonary hypertension.

**Figure 1:**
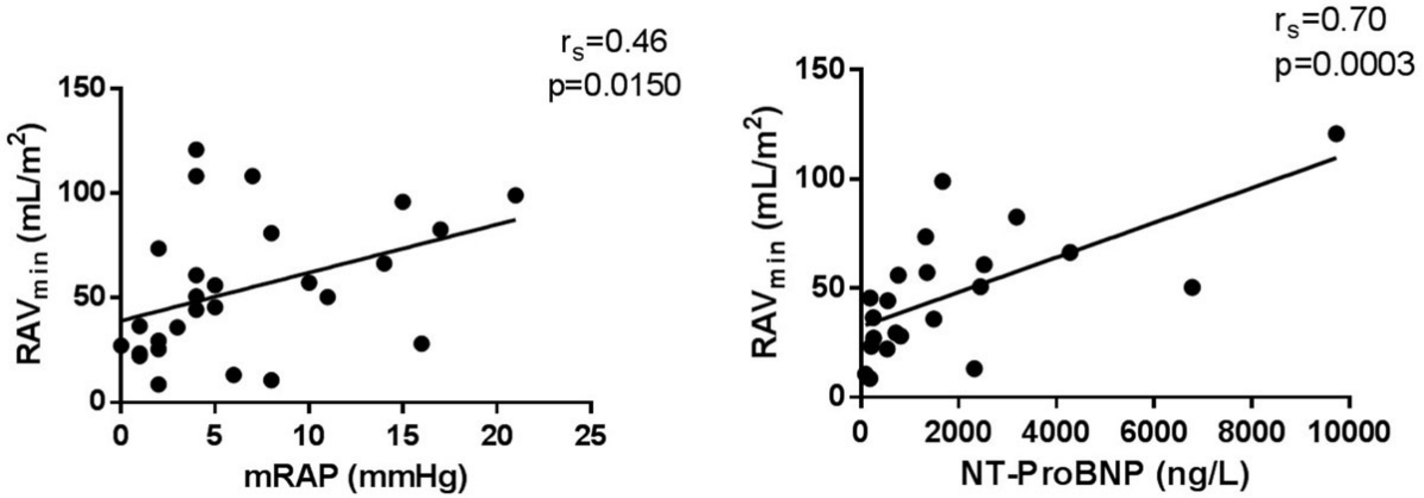
**The left panel shows the minimum right atrial volume (RAV**_**min**_**) compared to invasive mean right atrial pressure (mRAP), the right panel shows the RAV**_**min**_ **compared to NT-ProBNP**.

**Figure 2:**
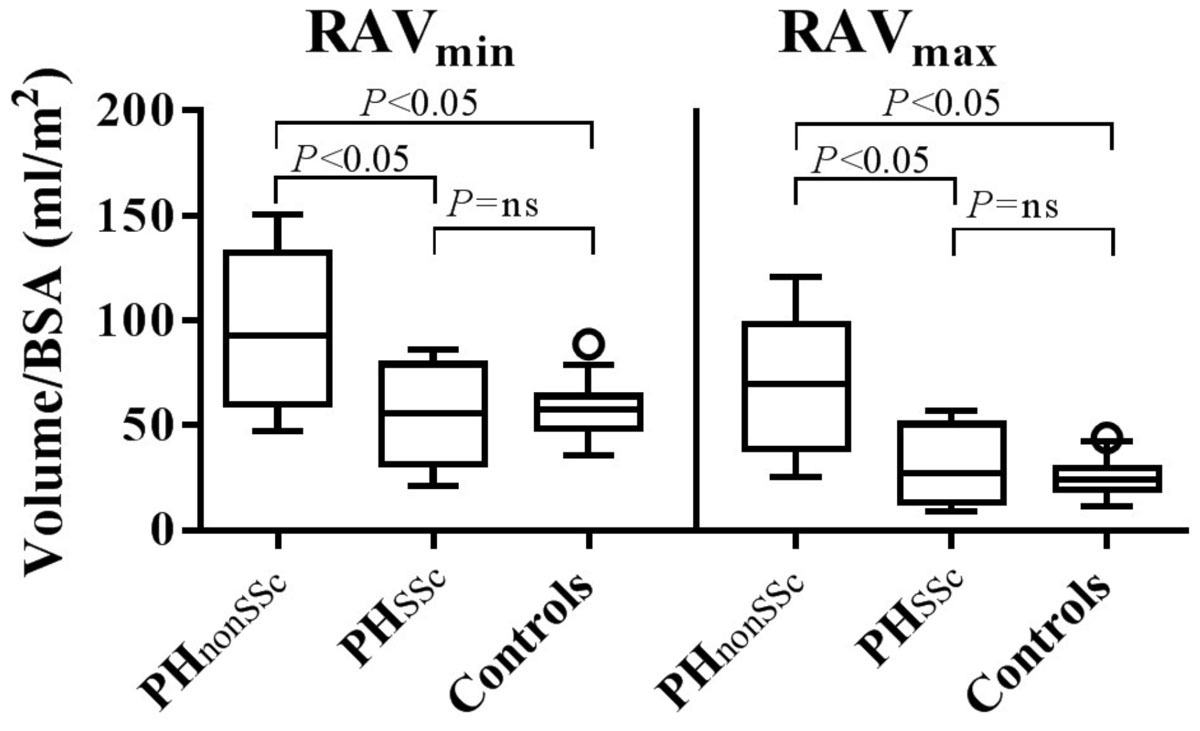
**Tukey boxplots of right atrial minimum volume (RAV**_**min**_**) and maximum volume (RAV**_**max**_**) in patients with precapillary PH without (PH**_**nonSSc**_**) and with Systemic Sclerosis (PH**_**SSc**_**) as well as in healthy controls**.

